# First Documented Human *Rickettsia aeschlimannii* Infection

**DOI:** 10.3201/eid0807.010480

**Published:** 2002-07

**Authors:** Didier Raoult, Pierre-Edouard Fournier, Philippe Abboud, François Caron

**Affiliations:** * Unité des Rickettsies, Université de la Méditerranée, Marseille, France; † Service de Maladies Infectieuses, Centre Hospitalier Universitaire de Rouen, Rouen, France

**To the Editor:** *Rickettsia aeschlimannii*, which was first isolated from *Hyalomma marginatum* ticks collected in Morocco in 1997 (1), has also been found in *H. marginatum* ticks from Zimbabwe, Niger, and Mali (2). For the past 3 years, we have included this species in the panel of rickettsiae for which sera from patients with suspected tickborne diseases are routinely tested. This procedure allowed us to document, by polymerase chain reaction (PCR) amplification and serologic testing, the first case of *R. aeschlimannii* human infection, which occurred in a patient returning from Morocco.

 This 36-year-old man traveled to Morocco in August 2000. On returning to France, he noticed a vesicular lesion of the ankle, which became necrotic and resembled the typical “tâche noire” of Mediterranean spotted fever (3). He became ill with fever of 39.5°C and a generalized maculopapular skin rash. Laboratory tests showed a normal blood cell count but moderately increased transaminases. An early serum specimen was tested to confirm the diagnosis of Mediterranean spotted fever. By microimmunofluorescence, the patient’s serum had immunoglobulin G and M titers of 1:32 and 1:16, respectively, against *R. aeschlimannii*; 0 and 1:16 against *R. conorii*, *R. africae*, *R. slovaca*, *R. helvetica*, and *R. massiliae*; and 0 and 1:8 against “*R. mongolotimonae*.” Western blot results showed that the patient’s serum reacted more intensively with *R. aeschlimannii* proteins than with those of the other tested rickettsiae. Attempted PCR amplification of a 630-nt portion of the rickettsial *ompA* gene (nt 70 to 701) (4) from the early serum specimen yielded a product of the expected size. The sequence of this amplicon allowed the identification of *R. aeschlimannii* with 100% homology. The patient was treated with doxycycline, 200 mg daily for 1 week, and rapidly recovered.

 This case is the first documented infection caused by *R. aeschlimannii,* a *Rickettsia* that had been isolated only from *Hyalomma marginatum* ticks from Africa. In our patient, its pathogenic role was demonstrated by PCR, a technique that has also proven useful in identifying other new rickettsial diseases, including infections with *R. helvetica* (5), *R. slovaca* (6), and *R. felis* (7). The serologic findings indicated antibodies at a higher level to *R. aeschlimannii* than to other tested species. *R. aeschlimannii* is phylogenetically distant from *R. conorii* but is closely related to *R. rhipicephali* and *R. montanensis*, which have never been described as human pathogens. This patient appeared to have a typical case of *R. conorii* infection, with seasonal and geographic characteristics favoring this diagnosis (3). This case was clinically and epidemiologically mistaken for *R. conorii* infection, suggesting that *R. aeschlimanii* may be another cause of Mediterranean spotted fever in Morocco.

 The systematic identification of rickettsial species in human infections continues to increase the number of recognized human pathogens (3). This finding has demonstrated once again that more than one species or serotype of tick-transmitted rickettsia may be prevalent in the same area, as observed, for example, with *R. slovaca, R. mongolotimonae*, and *R. conorii* in southern France (3); *R. africae* and *R. conorii* in sub-Saharan Africa (8); and *R. conorii* and Israeli spotted fever rickettsia in Sicily and Portugal (9). *Rickettsia* species first identified in ticks should be considered as potential human pathogens, as all recently described tick-transmitted rickettsiae pathogenic for humans were initially found in ticks and were considered nonpathogenic for several years (3).
